# Adding Insult on Injury: Immunogenic Role for Donor-derived Cell-free DNA?

**DOI:** 10.1097/TP.0000000000003240

**Published:** 2020-03-20

**Authors:** Shamik Dholakia, Iwijn De Vlaminck, Kiran K. Khush

**Affiliations:** 1 Oxford Transplant Center, Nuffield Department of Surgical Sciences, University of Oxford, Oxford, United Kingdom.; 2 Meinig School of Biomedical Engineering, Cornell University, Ithaca, NY.; 3 Division of Cardiovascular Medicine, Stanford University School of Medicine, Stanford, CA.

## Abstract

Donor-derived cell-free DNA (dd-cfDNA) in the blood circulation is an early marker of injury in solid organ transplantation. Here, we review recent evidence that indicates that dd-cfDNA may itself be a trigger of inflammation, thereby adding insult on injury. Early unresolving molecular allograft injury measured via changes in dd-cfDNA may be an early warning sign and may therefore enable stratification of patients who are at risk of subsequent allograft injury. Considering dd-cfDNA as a continuous and clinically significant biomarker opens up the potential for new management strategies, therapeutics, and ways to quantify interventions by considering the immunological potential of dd-cfDNA.

## BACKGROUND

Over the last decade, cell-free DNA (cfDNA) has been studied in the setting of various inflammatory diseases. Pathological processes such as atherosclerosis, primary Sjögren’s syndrome, rheumatoid arthritis, and systemic lupus erythematosus (SLE) are all characterized by inflammation in the presence of elevated cfDNA. Studies have also identified elevated cfDNA and inflammation in end-stage renal disease patients and in patients undergoing dialysis.^1,2^ In transplantation, donor-derived cfDNA (dd-cfDNA) has shown a significant association with clinical outcomes: dd-cfDNA is elevated in the presence of allograft injury and portends adverse posttransplant events such as estimated glomerular filtration rate decline, formation of de novo donor specific antibodies, and allograft rejection across many types of solid organ transplants.^3-5^

A systematic review by Knight et al^6^ considered 95 manuscripts on the topic of dd-cfDNA in transplantation, cataloging numerous studies that identified elevated levels of dd-cfDNA in the presence of allograft injury. These studies demonstrated elevated dd-cfDNA levels before and during acute rejection events, which returned to baseline following successful rejection treatment.^7,8^ It is now imperative, we consider emerging data that dd-cfDNA in organ transplant might itself be immunostimulatory.

In 1994, Polly Matzinger^9^ suggested a novel immunologic model, proposing that the immune system does not only identify between “self and nonself,” but also differentiates between “safe and dangerous” through recognition of pathogens or alarm signals from injured or stressed cells undergoing biochemical changes. In this way, the release of dd-cfDNA may act as a potential danger signal, thereby stimulating an immunological response, such as the activation of dendritic cells (DCs). With the activation of DCs, dd-cfDNA stimulation may perpetuate downstream signaling via mediators such as heat shock proteins, ATP, inflammatory cytokines (interferon gamma and interleukin [IL]-1b), and gene transcription, thereby adding insult to injury.^10,11^

### Importance of Innate Immunity

The innate immune system is the earliest host defense, whereby threats that overcome the physical and chemical barriers of the cellular epithelium lead to immediate immune recognition. This recognition includes cell-associated pattern recognition receptors (PRRs) such as tissue-residing macrophages and DCs.^11,12^ PRRs recognize lipids, lipoproteins, proteins, glycans and nucleic acids of viruses, parasites, bacteria, and fungi, which are generally referred to as pathogen-associated molecular patterns (PAMPs).^12,13^

**FIGURE 1.**
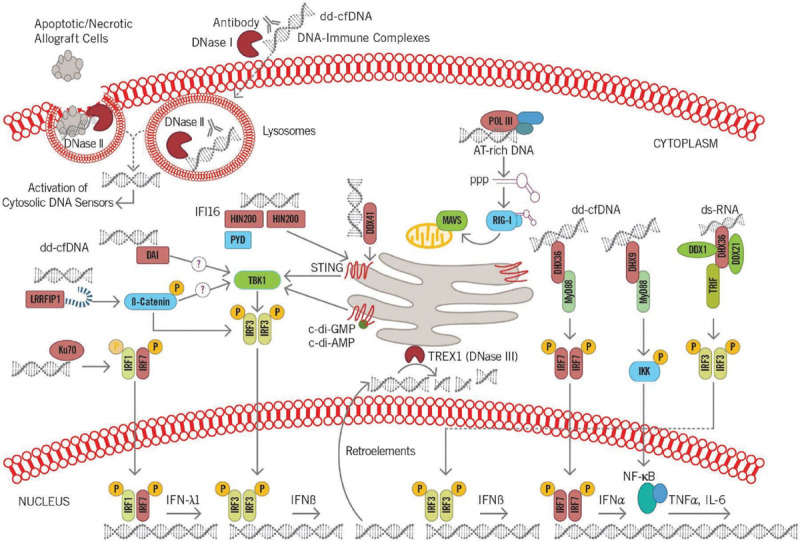
The transition from the innate immunity to adaptive immunity highlights the importance of how activation of the innate system may translate to larger immunological responses. c-di-AMP, c-di-adenosine monophosphate; c-di-GMP, cyclic diguanylate; DAI, DNA-dependent activator of interferon regulatory factor; dd-cfDNA, donor-derived cell-free DNA; DDX, dead-box families; DHX, deah-box families; DNase, deoxyribonuclease; ds-RNA, double-stranded RNA; HIN200, hemopoietic IFN-inducible nuclear proteins is a 200-amino acid motif; IFI16, gamma-interferon-inducible protein; IFN, interferon; IKK, IκB kinase; IL, interleukin; IRF, interferon regulatory factor; Ku70, protein; LRRFIP1, leucine-rich repeat (in Flightless 1) interacting protein 1; MyD88, myeloid differentiation factor 88; NF-κB, nuclear factor kappa B; POL III, RNA polymerase III; PPP, (E)-3-Phenyl-1-(2-pyrrolyl)-2-propenone; PYD, pyrin domain; RIG-I, retinoic-acid-inducible gene I; STING, stimulator of interferon genes; TBK1, TANK binding kinase 1; TNFα, tumor necrosis factor alpha; TREX1, 3 prime repair exonuclease 1; TRIF, TIR-domain-containing adapter-inducing interferon-β.

The archetype of selective pathogen recognition receptors, when considering PAMPs (which usually differ from host molecules), makes them well-suited for the initial discernment between nonself (pathogens) from noninfectious self.^14^ However, this construct is constantly being revisited, since PRRs also sense endogenous host molecules and commensal microbes, both of which can be released into the circulation from damaged tissue, and are known as danger- (or damage-) associated molecular patterns (DAMPs). The simultaneous recognition of PAMPs and DAMPs alerts the host immune system, triggering an ever more robust immune response (Figure 1).^15^

The innate immune system’s ability to identify RNA and DNA via endosomal and cytosolic PRRs is an indispensable mechanism that enables it to mount protective immune responses. This is important, particularly for detection of viruses, which often escape other methods of detection. Cytosolic DNA sensing has seen rapid evolution, with several new insights into mechanisms by which established PRRs, such as the retinoic-acid-inducible gene I like receptors and Toll-like receptors (TLRs), can engage with and identify microbial RNA.^16^

Considering the cross walking from microbial PAMP pathways to potential dd-cfDNA DAMP pathways results in the transplant recipient’s innate immune system with a potential plethora of receptors and mechanisms that may engage with the dd-cfDNA fragments released from the allograft, some of which are described here.

### Nucleic Acid-mediated Innate Immune Responses

Knowing that the various cfDNA types (double-stranded, single-stranded, and hypo-methylated) are respectively recognized by TLR3, TLR7, and TLR9 in mammals is an essential mechanism that can stimulate an immune response not controlled by the current immunosuppressive regimes typically used in the care of a transplant patient.

Elevated circulating mitochondrial DNA (mtDNA) is found in sepsis, trauma, and chronic organ-specific illnesses such as rheumatoid arthritis, SLE, and hepatitis. In transplantation, mtDNA is released into the circulation following reperfusion and also been shown to be a compelling danger signal that is recognized by the innate immune system, directly modifying the inflammatory response. An understanding of the pathways that may be activated by mtDNA is important, as the shared lineage between mtDNA and bacterial DNA suggest similarities that may be applicable to dd-cfDNA. The highlighting of a common nucleic acid mediate pathway of innate immunity represents an exciting new translational opportunity.^17^

## DEOXYRIBONUCLEASE AND NEUTROPHIL EXTRACELLULAR TRAPS

Allograft cell death, cell turnover, and biochemical changes all release cytosolic DNA into the circulation. An understanding of how dd-cfDNA may activate immune responses, either through cell surface receptor interaction or via intracellular signaling, is key when considering the activation of inflammatory and immune-stimulatory pathways. Acknowledging that dd-cfDNA has mechanisms to enter cells and activate intracellular pathways is important. Filev et al^18^ have shown spectrum green fluorescence-labeled oxidized cfDNA fragments are transferred into the cytoplasm of 80% of the cerebellum culture cells; meanwhile, the nonoxidized cfDNA fragments did not pass into the cells, in this way deoxyribonucleases (DNases) facilitate dd-cfDNA presentation through a variety of methods, and the potential in which dd-cfDNA is released or damaged as it enters the circulation may impact its antigenic potential and ability to enter cells.

In 2004, neutrophil extracellular traps (NETs) were described in defense pathway-inducing signals, whereby extracellular DNA (exDNA) is exported and assembled together with actin, histone, peroxidases, and other reactive oxygen species to develop a sticky matrix, called a NET.^19^ Importantly, proteins can be derived from NETs and serve as self-antigens mediating organ damage in autoimmune diseases through their role in the formation of immunostimulatory proteins.^20^

Clearance of degrading DNA that leaks from cells undergoing biochemical changes (or dead or dying cells) is thought to be controlled by the secreted DNases. Emerging studies have also shown evidence that DNases play a central role in which exDNase produced by bacteria, fungi, and other pathogens, as well as host tissue, typically breaks down NETs and DNA framework and thereby facilitates systemic dispersal and clearance of exDNA.^21,22^ DNases have also shown to prime DCs for alloantigen-specific CD4^+^ T cell proliferation and to promote human macrophage inflammatory cytokine production through activation of TLR.^23^

If NETs that form in transplantation contain dd-cfDNA, they potentially offer a further mechanism to which aberrant proteins derived from dd-cfDNA maybe be made and promote inflammation and innate immune activation, especially if these NETs are not cleared by DNAase.^24,25^

Neutrophils also carry key components of the complement alternative pathway such as properdin or complement factor P, complement factor B (CFB), and C3. However, CFB may further propagate the inflammatory response through further direction activation by dd-cfDNA.^26^ CFB circulates in the blood as a single chain polypeptide. Upon activation of the alternative pathway, it is cleaved by complement factor D. One of the active subunits is a serine protease, which is involved in the proliferation of preactivated B lymphocytes. CFB upregulation is independent of DNA-dependent activator of interferon regulatory factor (IRF), absent in melanoma 2 (AIM2), TLRs, and receptor for advanced glycation end products, but requires high-mobility group box (HMGB) proteins and myeloid differentiation factor 88.^27^

HMGB proteins are highly expressed in the nucleus but importantly are also present in the blood. HMGBs have a role in transcription and inflammation regarded as universal custodians for nucleic-acid-mediated innate immune responses. If CFB or HMGB proteins can be activated by dd-cfDNA fragments, it would support the importance that complement has shown in allograft rejection and how the donor material in the circulation may prime this response.^28^

## CYTOSOLIC DNA SENSORS

**FIGURE 2.**
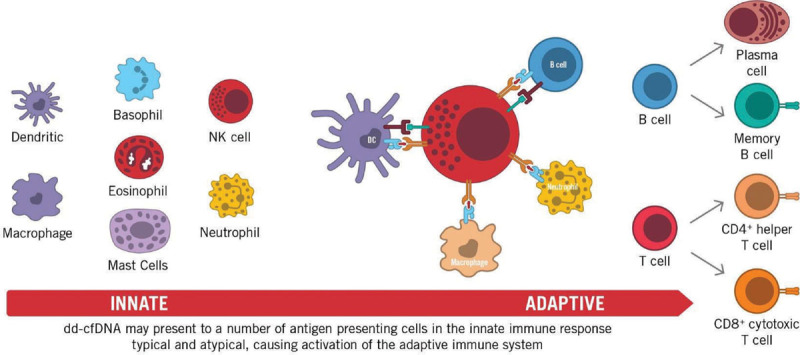
Donor-derived cell-free DNA (dd-cfDNA) being sensed by cytosolic DNAs sensors leads to the activation of distinct signaling pathways involved in recipient immune responses. These include activation of interferon regulatory factor (IRF) 3, IRF7, and nuclear factor kappa B (NF-κB) initiating the transcriptional induction of type I interferon genes and other proinflammatory genes such as interleukin-6 (IL-6) and tumor necrosis factor alpha (TNFα). DC, dendritic cell; NK, natural killer cell.

Cytosolic DNA of microbial or self-origin stimulates type I interferon production via the stimulator of interferon genes (STING)-TANK binding kinase 1 (TBK1)-IRF3 axis, as well as other proinflammatory cytokines (eg, IL-6 and tumor necrosis factor-alpha [TNF-α]), through activation of nuclear factor kappa B signaling and its effects on gene transcription.^27,29^ Figure 2 shows distinct cytosolic DNA sensors along with their select activating pathways.

Cytosolic DNA-sensing receptors, such as DNA-dependent activator of IRFs and AIM2, also activate the innate and adaptive immune systems. If dd-cfDNA is presented to these PRRs, the DNA-induced signaling pathway may converge on the STING adaptor and the kinase TBK1 either as a direct sensor or coactivator prompting further immunostimulation though cyclic di-nucleotides. Cyclic di-nucleotides are important secondary signaling molecules activated by DAMPs/PAMPs but also go on to activate the nucleotide-binding oligomerization domain (NOD)-like receptor protein 3 inflammasome.^30^

STING is activated by cyclic di-nucleotides called cyclic diguanylate and c-di-GMP/c-di-adenosine monophosphate and is then translocated to the Golgi apparatus, an event that triggers STING assembly with the downstream kinase TBK1. This then phosphorylates IRF3 to mediate downstream signaling events promoting transcriptional activation of inflammatory genes. As a result, many cells use distinct nucleases to eradicate both self and nonself DNA from extracellular space and cytosol such as DNases, phagolysosomes, 3 prime repair exonuclease 1 to attempt to avoid the detrimental effects of excess DNA-induced immune responses.^27,31^

Whether any of the immunosuppressant medications given in the posttransplant period inhibit any of the nucleases, potentially propagating the effect of dd-cfDNA, is a hypothesis actively being studied. One hypothesis considers aberrant removal of necrotic debris, a feature with inflammatory consequences in SLE. Lupus nephritis severity and higher cfDNA levels have been associated with lower urinary DNase I.^32^ In transplantation, tacrolimus inhibits Ca^2+^ dependent activation of nuclear factor kappa B (NFκB) and the nuclear factor of activated T cells in T lymphocytes, blocking T-cell receptor-mediated gene transcription, degranulation, exocytosis, and apoptosis. With DNase enzymes being Ca^2+^/Mg^2+^ dependent endonucleases, and prior studies demonstrating tacrolimus modulating calcium levels, the impact of immunosuppressive agents acting as potential DNase inhibitors should be further explored.^33^ If tacrolimus inhibits DNase, it may contribute to impaired ability to clear dd-cfDNA in transplant recipients and suggests a pathway similar to lupus nephritis whereby increased dd-cfDNA leads to the inflammatory signaling of donor material through NETs and TLRs.^26,34^

## SPHERICAL NUCLEIC ACIDS AND TOLL-LIKE RECEPTORS

Spherical nucleic acids are defined as structures that are an arrangement of densely packed, highly oriented nucleic acids in a spherical geometry. These tiny little balls of nucleic acids were demonstrated by Skakuj et al^35^ to important sequence-specific stimulators of antigen-presenting cells, through TLR9 regulated amplification of the downstream T-cell reaction in terms of both activation and proliferation.

TLR activation on DCs has also been shown to initiate signaling pathways via their signal adaptor protein, inducing translocation of the transcription factor NF-κB, and ultimately leading to dendritic maturation.^23^ This maturation is associated with significantly increased expression of costimulatory molecules as well as the secretion of pro-inflammatory cytokines such as IL-6. Subsequently, DCs migrate to lymph nodes, initiating an immune response that prompts naïve T cells to differentiate. This TLR induced migration mediates downregulation of receptors for inflammatory chemokines and upregulation of lymphoid chemokine receptors, especially CCR7.^36^ This is important in the context of transplantation, as many monoclonal antibody therapies used for induction or maintenance therapy may fail to deplete lymphocyte reservoirs.^37,38^

## HISTONES AND EXTRACELLULAR VESICLES

Extensive cell death releases chromatin components (dd-cfDNA, mtDNA, and histones) into the extracellular environment, all being potent antigenic stimulators. cfDNA and histones have been shown, both in vitro and in vivo, to display various immune-stimulatory effects. For example, histones induce cytotoxicity and proinflammatory signaling through TLR2 and TLR4, whereas cfDNA triggers signaling through intracellular nucleic acid-sensing mechanisms and TLR9.^39^ Histones have important proinflammatory functions upon their release from the nucleus into the extracellular environment and are likely to be released from allograft cells in the same manner as dd-cfDNA. Xu et al^40,41^ established that intravenous injection of histones into mice caused death with higher levels of TNF-α, and IL-6, whereas anti-histone antibodies reduced mortality in lipopolysaccharide, TNF-α, and puncture models of sepsis. Notably, the nucleosome (histone-DNA complex) is a TLR9-specific immune-stimulatory component that activates DCs.

Extracellular vesicles (EVs) release encapsulated content that often containing inflammatory material, such as histones, mtDNA, and cfDNA and are key in mediating immune regulation and intercellular communication through their role in immune suppression, antigen presentation, antitumor immunity, and autoimmunity. EV degradation pathway is based upon the autophagy-lysosome pathway, making circulating leucocytes integral to the clearance of EV. Having impaired immunity as in transplantation may lead to this inflammatory debris failing to clear efficiently activating pathways associated with inflammation.^42^

## THE TRANSPLANT INFLAMMASOME

**Figure 3.**
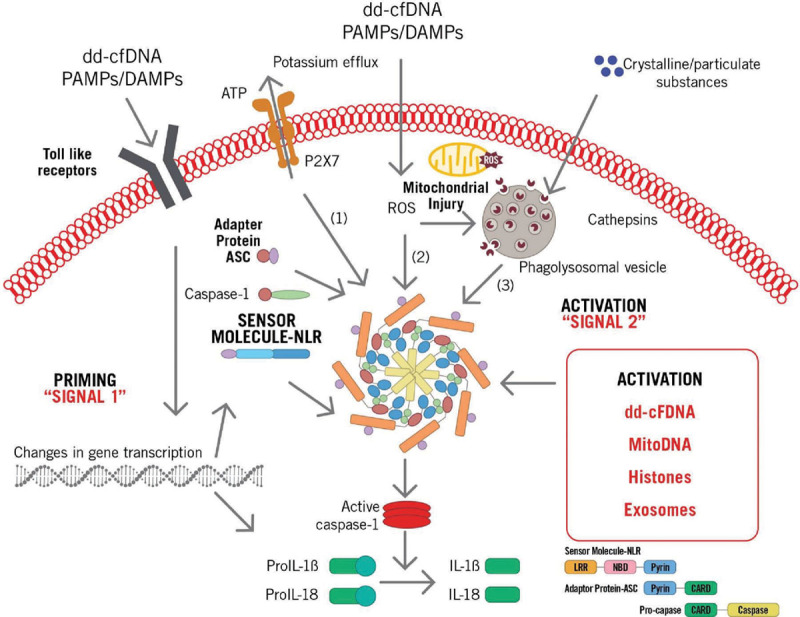
Donor-derived cell-free DNA (dd-cfDNA) driven inflammasome assembly via pathogen-associated molecular pattern (PAMP), damage-associated molecular pattern (DAMP) signaling via Toll-like receptors (TLRs) and other interactions leading to caspase-1 activation and proteolytic cleavage of both pro–interleukin (IL)-1β and pro-IL-18 into biologically active, mature forms IL-1β and IL-18. ASC, apoptosis-associated speck-like protein; CARD, caspase activation and recruitment domain; LLR, leucine-rich repeat; mitoDNA, mitochondrial DNA; NBD, nucleotide-binding domain; NLR, nucleotide-binding oligomerization domain-like receptor; ROS, reactive oxygen species.

Komada et al^43^ have shown that macrophage uptake of necrotic cell DNA activates the AIM2 inflammasome to regulate a proinflammatory phenotype in chronic kidney disease. Inflammasomes are large molecular weight cytosolic complexes that regulate the activation of caspases. There are several types of inflammasomes, and each is activated by a unique PRR response. Two signals (priming and activation) are characteristically involved in inflammasome activation and are important in transplantation (Figure 3).

Signal one (priming signal) involves the recognition of DAMPs and PAMPs, such as dd-cfDNA or microbes, which interact with TLRs and induce downstream gene transcription, producing pro–IL-1β. This is followed by signal 2 (activation signal), which involves additional acknowledgment of PAMPs and/or DAMPs, such as dd-cfDNA as well as other molecules (such as uric acid) that activate the pathway via NOD-like receptor protein 3. Signal 2 promotes caspase-1–dependent cleavage of pro–IL-1β, which produces IL-1β and pyroptosis.^44^

NOD-like receptors (NLRs) contribute to inflammasome activation and are classified as an intracellular family of pathogen recognition molecules that also independently activate the nuclear factor kappa B complex, leading to expression of proinflammatory and chemotactic cytokines. NLRs are characterized by a NOD and ligand-recognizing leucine-rich repeats. NLRs recognize a number of toxins, flagellin, and peptidoglycan, as well as aberrant proteins formed by cfDNA; activation of the inflammasome through dd-cfDNA signaling thus has many potential mechanisms.^45^

Inflammasome activation causes the release of multiple proinflammatory cytokines. Early insults to the allograft, including ischemia-reperfusion injury during organ procurement and implantation, infections associated with immunosuppression, and allograft rejection all release both PAMPs and DAMPs. Inflammasome activation and IL-1 expression may cause upregulation of chemokines and adhesion molecules, which may promote allograft mononuclear phagocyte recruitment, neutrophil sequestration, and T cell activation, all of which are critical steps in the pathway from acute allograft insult to chronic allograft dysfunction and ultimately allograft loss.^46^

## DISCUSSION

In summary, a growing number of studies point to a potential role for dd-cfDNA beyond assessment of acute injury, showing that dd-cfDNA may itself be a potential trigger of inflammation, thereby adding insult to injury. It has been well described that patients with early unresolving allograft injury, as measured via % dd-cfDNA, are at risk of subsequent allograft dysfunction. Thus, consideration of dd-cfDNA levels as a continuum imparts value to changing levels over time, rather than considering absolute thresholds. Indeed, following levels of dd-cfDNA over time may provide windows of opportunity to intervene (for instance by augmenting immunosuppression to prevent acute rejection) before the occurrence of adverse events. This approach may enable clinicians to take a proactive rather than reactive approach to posttransplant patient management.

Continued examination and use of dd-cfDNA as a surveillance tool will help to further uncover innate immune mechanisms and will provide enhanced understanding to aid in the identification of the “tipping point” between true immune quiescence and recipient immune activation. As current immunosuppressive agents do not target innate immune effectors, such TLRs and the inflammasome, the potential to develop novel drugs to better prevent immune-mediated transplant complications is an intriguing possibility.
